# Worries, beliefs and factors influencing perinatal COVID-19 vaccination: a cross-sectional survey of preconception, pregnant and lactating individuals

**DOI:** 10.1186/s12889-022-14617-4

**Published:** 2022-12-23

**Authors:** Serine Ramlawi, Katherine A. Muldoon, Sandra I. Dunn, Malia S. Q. Murphy, Alysha L. J. Dingwall-Harvey, Ruth Rennicks White, Romina Fakhraei, Shi Wu Wen, Mark C. Walker, Deshayne B. Fell, Tali Bogler, Darine El-Chaâr

**Affiliations:** 1 Clinical Epidemiology Program, Ottawa Hospital Research Institute, Ottawa, Canada; 2 Department of Obstetrics and Gynecology, Faculty of Medicine, University of Ottawa, Ottawa, Canada; 3 School of Nursing, University of Ottawa, Ottawa, Canada; 4 Department of Obstetrics, Gynaecology and Newborn Care, The Ottawa Hospital, Ottawa, Canada; 5 School of Epidemiology and Public Health, Faculty of Medicine, University of Ottawa, Ottawa, Canada; 6 Children’s Hospital of Eastern Ontario Research Institute, Ottawa, Canada; 7 Department of Family and Community Medicine, St. Michael’s Hospital, Toronto, Canada; 8 Department of Family and Community Medicine, University of Toronto, Toronto, Canada; 9 Li Ka Shing Knowledge Institute, St. Michael’s Hospital, Toronto, Canada; 10 The Ottawa Hospital, General Campus, CPCR, 501 Smyth Rd, Box 241, Ottawa, ON K1H 8L6 Canada

**Keywords:** COVID-19, SARS-CoV2, COVID-19 vaccine, Vaccine acceptance, Pregnancy, Maternal child health, Lactating

## Abstract

**Background:**

COVID-19 vaccines are recommended for pregnant and lactating individuals, and there is substantial evidence for their safety and effectiveness. As the pandemic continues, information on worries and beliefs surrounding perinatal COVID-19 vaccination remains important to inform efforts aimed at improving vaccine uptake. Our objectives were to assess factors associated with COVID-19 vaccination among perinatal individuals; and to explore motivational factors associated with willingness to be vaccinated among unvaccinated perinatal individuals.

**Methods:**

This was a cross-sectional web-based survey of preconception, pregnant, and lactating individuals in Canada. The outcomes of interest were vaccination with at least one dose of any COVID-19 vaccine and willingness to be vaccinated among unvaccinated individuals. Sample characteristics were summarized using frequencies and percentages. The association between eight prespecified risk factors and two outcomes (vaccination status and willingness to be vaccinated) was assessed by logistic regression. Odds ratios (OR) and 95% confidence intervals (CI) were calculated for the total sample, and across perinatal sub-groups.

**Results:**

Among 3446 survey respondents, there were 447 (13.0%) preconception, 1832 (53.2%) pregnant, and 1167 (42.4%) lactating. There were 1460 (42.4%) and 1982 (57.5%) who were vaccinated and unvaccinated, respectively. Factors positively associated with COVID-19 vaccine status were speaking to a healthcare provider about vaccination during the perinatal period (aOR:2.35, 95% CI:1.97–2.80) and believing that the COVID-19 vaccine is effective (aOR:1.91, 95% CI:1.46–2.48). Factors negatively associated with vaccine status included worries about fetal growth and development (aOR:0.55, 95% CI:0.43–0.70) and future child behavioral/neurodevelopmental problems (aOR:0.59, 95% CI:0.46–0.75). Among unvaccinated individuals specifically, characteristics positively associated with willingness to vaccinate were speaking to a healthcare provider (aOR:1.67, 95% CI:1.32–2.12) and believing the COVID-19 vaccine is effective (aOR:3.56, 95% CI:2.70–4.69). Factors negatively associated with willingness were concerns over infertility (aOR:0.66, 95% CI:0.49–0.88), fetal growth and development (aOR:0.33, 95% CI:0.24–0.46), and future child behavioral/neurodevelopmental problems (aOR:0.64, 95% CI:0.48–0.84).

**Conclusions:**

In this Canadian perinatal population, approximately 42% reported COVID-19 vaccination. Among unvaccinated individuals, willingness to receive vaccination was high (73%). Factors enhancing vaccine willingness included discussions with healthcare providers and believing the vaccine was effective. Concerns regarding vaccine safety, particularly with respect to fetal/child development, were the greatest barriers to vaccine uptake.

**Supplementary Information:**

The online version contains supplementary material available at 10.1186/s12889-022-14617-4.

## Background

Pregnant individuals are at higher risk of complications associated with COVID-19 infection including intensive care unit admission, invasive ventilation, preterm birth, hypertensive disorders of pregnancy, and maternal and neonatal morbidity and mortality [1–3]. Initially it was recommended that individuals should not receive the COVID-19 vaccine during pregnancy or lactation, however amid increasing evidence for the substantial risks of SARS-CoV-2 infection during pregnancy, pregnant individuals were later declared a high-priority population for vaccination [4–6].

In a large, Canadian population-based study of COVID-19 vaccination during pregnancy of over 97,000 individuals, it was found that 23% received at least one COVID-19 vaccine dose during pregnancy, 46% were vaccinated after pregnancy, and 31% were never vaccinated [7]. When compared to unvaccinated pregnant individuals, there was no significant risk in adverse obstetrical outcomes (e.g., postpartum hemorrhage, cesarean delivery) or infant outcomes (e.g., chorioamnionitis, intensive care admission, or low APGAR score) [7]. Several other studies have also shown that receiving COVID-19 vaccines during pregnancy does not increase risk to the pregnant individual or fetus; however, vaccine coverage among pregnant individuals remains below women of reproductive age in the general population. In Canada, coverage estimates for vaccines routinely recommended during pregnancy, including influenza and tetanus-diphtheria-acellular pertussis (Tdap), have been estimated at 45% and 42%, respectively [8], with wide variation across provinces and territories [9]. Perinatal vaccination programs continue to explore strategies to encourage vaccine confidence and uptake [10], particularly as the COVID-19 pandemic continues and additional doses of COVID-19 vaccines are recommended.

The rapid expansion of COVID-19 vaccine eligibility criteria for high-risk populations, combined with a deluge of mainstream media coverage, conflicting or misinformation on social networks, and other factors related to vaccine acceptance and hesitancy confounded early COVID-19 vaccine decision-making for new and expectant families [11, 12]. Individual factors associated with vaccine hesitancy during pregnancy include: believing rumors and misinformation from social media, accessing information from non-credible sources [13, 14], lack of knowledge about the burden of disease from COVID-19, and lack of information and access to vaccines from trusted healthcare providers (HCP) [15]. Environmental and social contextual factors that have been associated with lower acceptance of COVID-19 vaccination for pregnant individuals include non-white ethnicity, as well as lower income, education, employment status and maternal age [7, 13, 16–18]. Other factors associated with lower acceptance of COVID-19 vaccination during pregnancy include fears or worries about safety of vaccines, long-term side effects of the COVID-19 vaccine and adverse reactions [13, 17], not believing COVID-19 exists, and mistrust of the health system and in vaccines [13, 17].

Only with a thorough understanding of individual perceptions, contextual factors, and facilitators and barriers to COVID-19 vaccination in the pregnant and lactating population can vaccine acceptance efforts be effectively tailored to reduce hesitancy, ensure informed decision-making and optimize uptake. This is important as the COVID-19 pandemic continues and vaccine boosters continue to be recommended. The objectives of this study were to: 1) assess factors associated with vaccination status among preconception, pregnant and lactating individuals; and 2) explore individual motivational factors associated with willingness to be vaccinated among unvaccinated preconception, pregnant and lactating individuals.

## Methods

### Study design and sample

This was a cross-sectional online survey among preconception (defined as either actively trying to conceive or planning to become pregnant in the next 12 months), pregnant and lactating individuals in Canada. Respondents had to be able to read in English and have access to the internet to complete the survey. Data collection occurred from March 29^th^ until August 12^th^, 2021. By May 2021, the National Advisory Committee on Immunization (NACI) in Canada began recommending COVID-19 vaccination for all pregnant and lactating individuals; prior to this, it was recommended only for pregnant individuals who were eligible due to being in an earlier high priority group (e.g., HCP).

### Survey development

The survey was developed in consultation with content experts (vaccine and perinatal epidemiologists, and maternity HCP), informed by a review of the scientific literature, and other vaccine surveys of the perinatal population. The survey was designed with gender-inclusive language for childbearing individuals of all gender identities. We pilot-tested the survey over a one-week period in a sample of 552 respondents and adapted the questions as needed. Informed consent to participate was implied upon participant submission of their survey responses. Responses were anonymous with no personal health information collected. Study data were stored on secure institutional servers to ensure privacy and protection. Survey questions are included in Additional File 1.

### Recruitment and data collection

The survey was hosted on LimeSurvey (Version 2.59.1 + 170,116) and advertised online on a study-specific webpage, and locally in Ottawa, Canada, at The Ottawa Hospital Department of Obstetrics and Gynaecology and the Monarch Centre, a multidisciplinary maternal and newborn health clinic. Links to the survey were posted on Canadian social media accounts and could be shared publicly to facilitate snowball sampling. Survey completion was voluntary, and no incentives were offered.

This was a convenience sample using an open and web-based survey, with a total of 79 survey items using adaptive questioning. Respondents were provided with non-response options such as “prefer not to answer” or “not applicable” and had the option to skip questions. Respondents had the ability to review and change their answers before submitting the survey.

Multiple entries from the same individual were prevented using cookies. Participants using the same device (assuming they have not cleared their cache and cookies) would be notified that they have previously submitted the survey. The survey did not log IP addresses of respondents and log file analysis was not used. The view rate and participation rate were not calculated as the online survey was widely shared through social media outlets and local advertisements.

### Study outcomes and measures

The primary outcome of interest was COVID-19 vaccination status, which was defined as having received at least one dose of any COVID-19 vaccine. The secondary outcome of interest was willingness to be vaccinated in their current perinatal state (preconception, pregnant, lactating) among unvaccinated individuals.

Respondents’ attitudes towards COVID-19 vaccines were captured using questions about their worries and beliefs about the vaccines. Vaccinated individuals were asked about their reasons for getting vaccinated and unvaccinated respondents were asked about factors that would motivate them to get vaccinated. Respondents were asked about their main sources of COVID-19 information.

Socio-demographic information collected included age, race/ethnicity, sexual orientation, marital status, primary language, education, and income. Health related history included any pre-existing health conditions and information on COVID-19 infection.

### Data analysis

Characteristics of the sample were summarized using frequencies and percentages. Two sets of analyses were conducted for each outcome. The first was to assess the factors associated with vaccination status among the total sample. The second was restricted to unvaccinated respondents, to assess factors associated with willingness to be vaccinated. The analyses were run in each sample, and then stratified by each of the reproductive status sub-groups (preconception, pregnant, lactating).

To investigate factors most strongly associated with vaccination status and the willingness to be vaccinated among the unvaccinated, we conducted logistic regression models in each sub-group to generate odds ratios (OR) and 95% confidence intervals (CI). We included the eight variables in the multivariable logistic regression models: four different worries (i.e. may lead to infertility, pregnancy loss or stillbirth, problems with fetal growth and development, problems with future behavioral neurodevelopmental problems for child), two different beliefs (i.e. has a good understanding of how the COVID-19 vaccines works, COVID-19 vaccine is effective), spoken to a HCP about vaccines during the perinatal period, and the date that the survey was completed, to account for variability in the availability of the vaccine or changes to COVID-19 related policies. Analyses were conducted using SAS 9.4.

This study received approval from the Ottawa Health Science Network Research Ethics Board [20210167-01H].

## Results

### Participant characteristics

**Fig. 1:**
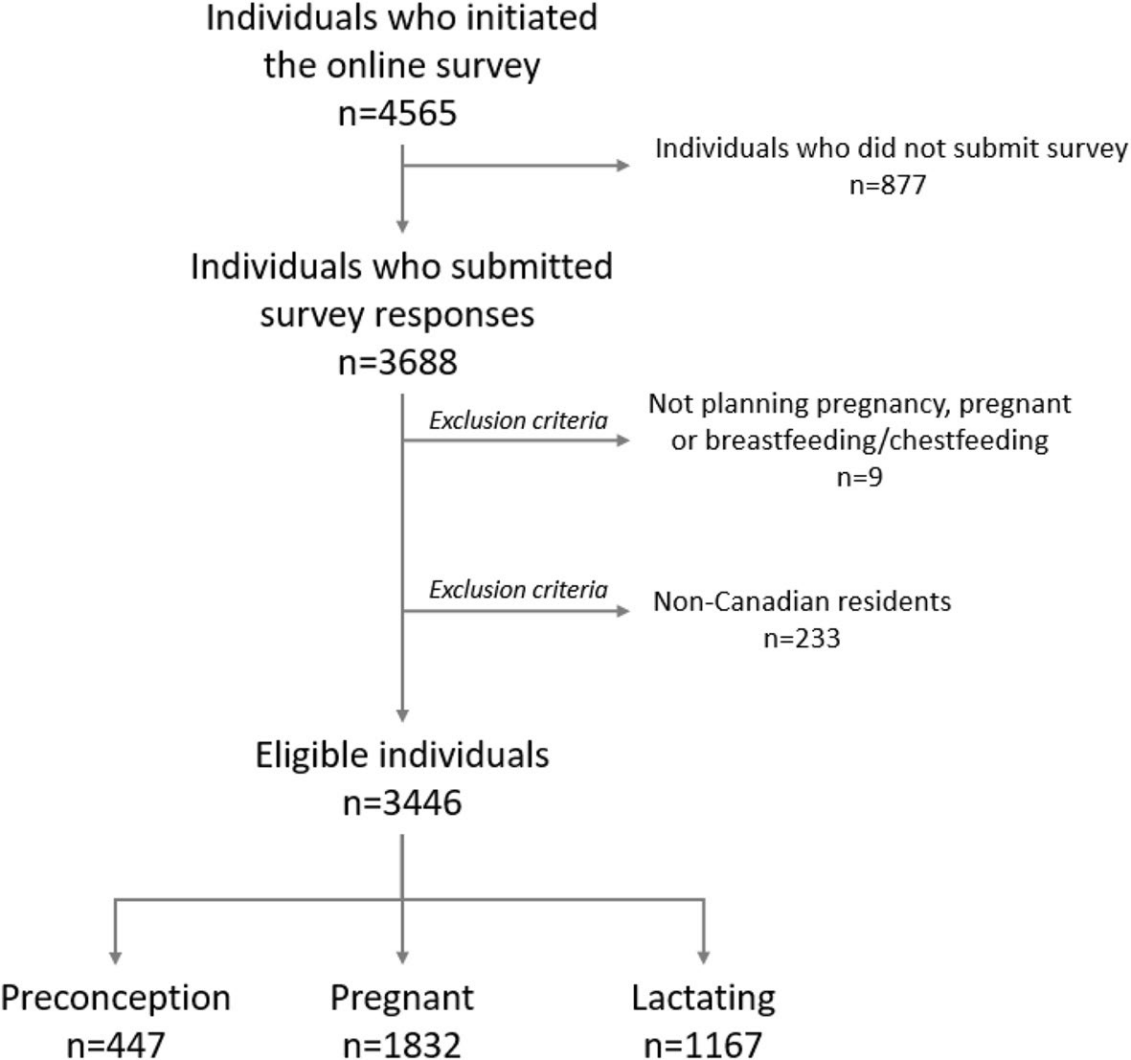
Flowchart for sample selection

Between March 29^th^ and August 12^th^, 2021, 4565 individuals started the survey. Of those, 3688 individuals submitted the survey for a completion rate of 80.8%. We excluded 242 responses: 9 were not eligible, and 233 resided outside of Canada. A total of 3446 unique survey responses were used in the final analyses (Fig. 1).

Table 1 displays the descriptive characteristics of survey respondents. The sample included 447 (13.0%) preconception, 1832 (53.2%) pregnant, and 1167 (42.4%) lactating individuals. There were 1460 (42.4%) vaccinated individuals, with the most common vaccine product being Comirnaty mRNA vaccine (88.4% of vaccinated sample). The majority of respondents were 30–39 years (80.4%), reported White race/ethnicity (83.0%) and with a household incomes above $120,000 CAD (65.8%). The most frequently reported pre-existing health conditions were obesity (11.8%), respiratory conditions (9.8%), and severe allergies (5.4%).

**Table 1:** Descriptive characteristics of survey participants (*n* = 3446) who were either preconception, pregnant or breastfeeding

Variables	n	%
Reproductive status		
Preconception	447	(13.0)
Pregnant	1832	(53.2)
Breastfeeding	1167	(33.9)
Age (years)		
18–29	568	(16.5)
30–39	2769	(80.4)
40–49	109	(3.16)
Race or Ethnicity		
White	2860	(82.99)
Asian	267	(7.75)
Latin American	54	(1.57)
Middle Eastern	40	(1.16)
Black	32	(0.93)
First Nations	26	(0.75)
Another race or ethnicity^a^	167	(4.85)
Sexual orientation: Heterosexual vs other^b^	3295	(95.6)
Marital status: Married/Common Law vs other	3399	(98.6)
Primary language: English vs other^c^	3313	(96.1)
Education:		
Bachelor’s degree or above	2962	(86.0)
Below Bachelor’s degree^d^	459	(13.3)
Prefer not to answer	25	(0.7)
Gross household income (CAD)^e^		
$120,000 +	2268	(65.8)
$90,000-$119,999	497	(14.4)
$60,000—$89,999	266	(7.72)
Below $60,000	92	(2.7)
Prefer not to answer	323	(9.4)
Occupation: Health care provider^f^	912	(26.5)
Any pre-existing health condition^g^		
Diabetes (Type 1, Type 2, gestational)	76	(2.21)
Hypertension	59	(1.71)
Obesity	407	(11.8)
Respiratory conditions	339	(9.84)
Severe allergies	185	(5.37)
Heard about the survey from social media	3216	(93.3)
COVID-19 information sources (select any)		
Government issued websites	3192	(92.6)
Health care provider	2748	(79.7)
News broadcasting	2483	(72.0)
Social media	1783	(51.7)
Pregnancy and breastfeeding professional societies	1411	(41.0)
General pregnancy and childbirth/parenting websites	269	(7.81)
Self-reported history of COVID-19 infection	106	(3.08)
Spoke to HCP about COVID-19 vaccine during perinatal period	2252	(65.4)
Vaccinated (*n* = 1460)^h^	1460	(42.4)
If yes, how many doses		
One	1290	(88.4)
Two	170	(11.6)
If yes, which brand did you receive at least one dose of		
Comirnaty mRNA vaccine	1295	(88.70)
Moderna mRNA vaccine	293	(20.1)
AstraZeneca Oxford vaccine	23	(1.58)
Unvaccinated (*n* = 1982)		
Willing to be vaccinated	1443	(72.8)

^a^Other race/ethnicity groups include Mixed heritage and prefer not to answer

^b^Other sexual orientations include homosexual, gay, lesbian, queer, bisexual, pansexual, asexual, prefer not to answer

^c^Other languages include Arabic, Mandarin, Cantonese, French, Italian, Portuguese, Spanish. All other listed languages had less than 5 counts

^d^Education: Below a Bachelors degree includes: College, CEGEP (Collège d'enseignement général et professionnel), high school, high school equivalency, trades certificate, university certificate or diploma below the bachelors level

^e^For reference, Canadian median household income for families is 98,690 CAD in 2019

^f^Health care providers include physician, nurse, allied health, paramedic/first responder, community health worker, traditional healer, and other health care provider

^g^Respondents may select multiple answers

^h^Vaccination questions use 1460 vaccinated participants as the denominator

**Fig. 2:**
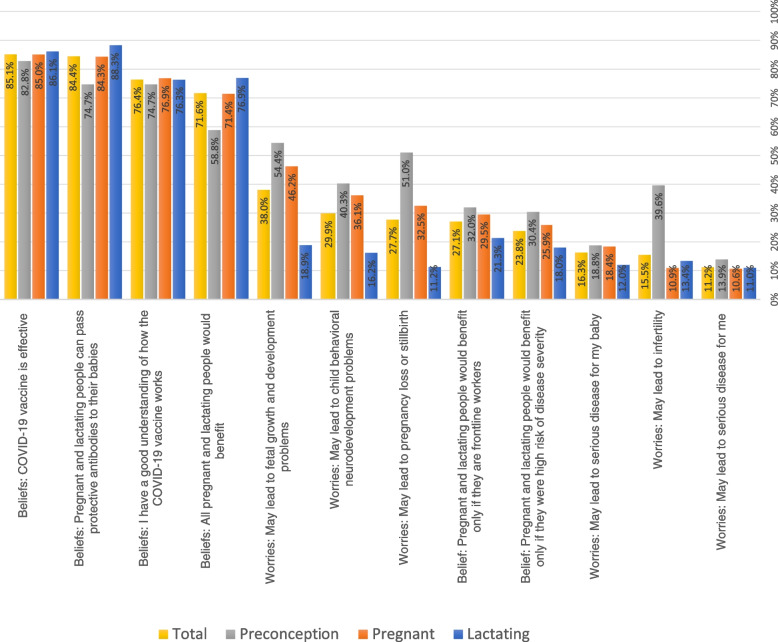
Worries and beliefs about the COVID-19 vaccine among preconception, pregnant, and breastfeeding people (*n* = 3446). NB: Percentages are calculated within each sub-group; Preconception subgroup = 447; Pregnant sub-group = 1832; Lactating sub-group = 1167

Figure 2 displays the most common participant worries and beliefs about COVID-19 vaccines, stratified by reproductive status. In the total sample, the most common worries were those related to fetal growth and development (38.0%), future behavioural and neurodevelopmental problems in the child (29.9%), and pregnancy loss (27.7%). The most common beliefs were that COVID-19 vaccines are effective (85.1%), and that pregnant and lactating people could pass antibodies to their babies (84.4%).

**Fig. 3:**
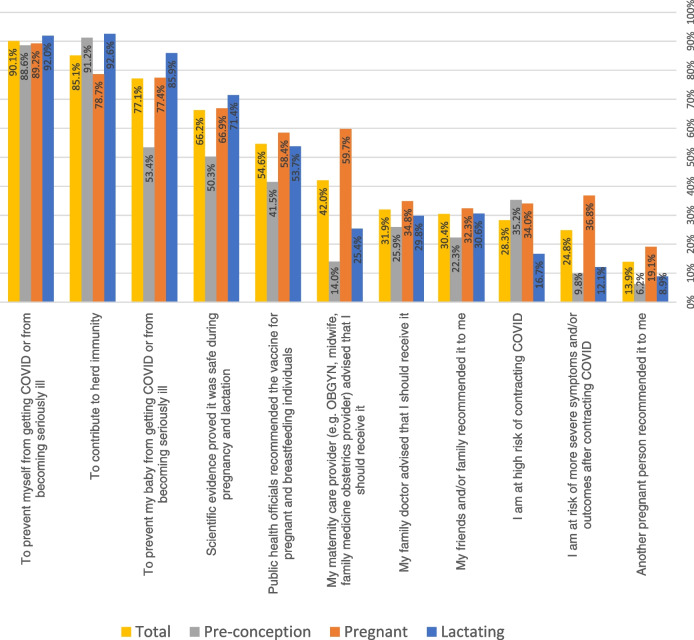
Reasons for getting vaccinated among vaccinated preconception, pregnant, and breastfeeding people (*n* = 1460). NB: Percentages are calculated within each vaccinated sub-group; Preconception subgroup = 193; Pregnant sub-group = 770; Lactating sub-group = 497. *NB: Preconception—protecting fetus/baby (planned future pregnancy baby)

Figure 3 displays reasons why vaccinated respondents chose to get vaccinated, stratified by reproductive status. In the total sample, the most common reasons were to prevent themselves from getting COVID-19 or from becoming seriously ill (90.1%), to contribute to herd immunity (85.1%) and to prevent their baby from getting COVID-19 (77.1%).

**Fig. 4:**
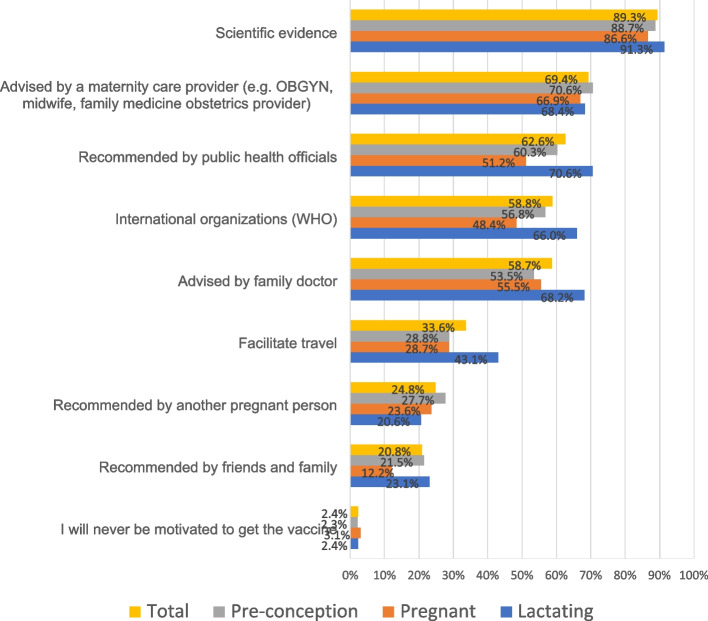
Motivating factors to get vaccinated among unvaccinated preconception, pregnant, and breastfeeding people (*n* = 1982). NB: Percentages are calculated within each unvaccinated sub-group; Preconception subgroup = 254; Pregnant sub-group = 1062; Lactating sub-group = 670

Figure 4 displays motivating factors to get vaccinated among the unvaccinated group, stratified by reproductive status. The most common motivating factors were scientific evidence (89.3%), being advised by a maternity care professional to get the vaccine (69.4%), and if it was recommended by public health officials (62.6%).

Table 2 displays factors associated with COVID-19 vaccination stratified by reproductive status. In the total sample, the factors positively associated with COVID-19 vaccination were speaking to a HCP about the vaccine during the perinatal period (adjusted OR [aOR]: 2.35, 95%CI: 1.97–2.80) and believing that COVID-19 vaccines are effective (aOR: 1.91, 95%CI: 1.46–2.48). The factors negatively associated with vaccination were worries about fetal growth and development (aOR: 0.55, 95%CI: 0.43–0.70) and future child behavioral neurodevelopmental problems (aOR: 0.59, 95%CI: 0.46–0.75). Similar patterns were found across each sub-group.

**Table 2:** Factors associations with COVID-19 vaccination status (*n* = 3446) and sub-group analyses among preconception, pregnant, and breastfeeding individuals

**A. Total (*n* = 3446)**	**Vaccinated**		**OR (95% CI)**	**aOR (95% CI)**
Variables	Yes = 1460	No = 1986		
	n (%)	n (%)		
Spoken to HCP about vaccine during perinatal period	1149 (78.7)	1103 (55.5)	2.96 (2.54–3.45)	2.35 (1.97–2.80)
Beliefs: Understand how vaccine works	1228 (84.11)	1405 (70.8)	2.19 (1.85–2.60)	1.55 (1.26–1.92)
Beliefs: Believe vaccines are effective	1333 (91.3)	1600 (80.56)	2.53 (2.05–3.13)	1.91 (1.46–2.48)
Worries: Infertility	134 (9.2)	399 (20.1)	0.40 (0.33–0.50)	0.81 (0.61–1.07)
Worries: Pregnancy loss	255 (17.5)	700 (35.3)	0.39 (0.33–0.46)	0.70 (0.55–0.88)
Worries: Fetal growth and development problems	372 (25.5)	938 (47.2)	0.38 (0.33–0.44)	0.55 (0.43–0.70)
Worries: Child behavioural neurodevelopmental problems	286 (19.6)	745 (37.5)	0.41 (0.35–0.48)	0.59 (0.46–0.75)
Date of survey completion^a^			1.04 (1.04–1.05)	1.04 (1.04–1.05)
**B. Pre-conception (*n* = 447)**	**Vaccinated**		**OR (95% CI)**	**aOR (95% CI)**
Variables	Yes = 193	No = 254		
	n (%)	n (%)		
Spoken to HCP about vaccine during perinatal period	104 (53.9)	86 (33.9)	2.28 (1.55–3.35)	2.45 (1.54–3.90)
Beliefs: Understand how vaccine works	168 (87.1)	166 (65.4)	3.56 (2.18–5.83)	1.60 (0.89–2.88)
Beliefs: Believe vaccines are effective	178 (92.2)	192 (75.6)	3.83 (2.10–6.98)	2.56 (1.26–5.20)
Worries: Infertility	50 (25.9)	127 (50.0)	0.35 (0.23–0.52)	0.67 (0.38–1.17)
Worries: Pregnancy loss	65 (33.7)	163 (64.2)	0.28 (0.19–0.42)	0.68 (0.39–1.20)
Worries: Fetal growth and development problems	62 (32.1)	181 (71.3)	0.19 (0.13–0.29)	0.44 (0.24–0.83)
Worries: Child behavioural neurodevelopmental problems	37 (19.2)	143 (56.3)	0.18 (0.12–0.29)	0.47 (0.25–0.89)
Date of survey completion			1.02 (1.01–1.03)	1.02 (1.01–1.03)
**C. Pregnancy (*n* = 1832)**	**Vaccinated**		**OR (95% CI)**	**aOR (95% CI)**
	Yes = 770	No = 753		
Variables	n (%)	n (%)		
Spoken to HCP about vaccine during perinatal period	714 (92.7)	802 (75.5)	4.13 (3.04–5.61)	2.65 (1.87–3.77)
Beliefs: Understand how vaccine works	655 (85.1)	753 (70.9)	2.33 (1.84–2.97)	1.72 (1.28–2.30)
Beliefs: Believe vaccines are effective	696 (90.4)	862 (81.2)	2.18 (1.64–2.90)	1.44 (1.01–2.07)
Worries: Infertility	38 (4.9)	162 (15.3)	0.29 (0.20–0.42)	0.67 (0.42–1.06)
Worries: Pregnancy loss	145 (18.8)	451 (42.5)	0.31 (0.25–0.39)	0.54 (0.40–0.74)
Worries: Fetal growth and development problems	243 (31.6)	604 (56.9)	0.35 (0.29–0.43)	0.59 (0.43–0.89)
Worries: Child behavioural neurodevelopmental problems	191 (24.8)	471 (44.4)	0.41 (0.34–0.51)	0.62 (0.46–0.85)
Date of survey completion			1.04 (1.04–1.05)	1.05 (1.04–1.05)
**D. Breastfeeding (*n* = 1167)**	**Vaccinated**		**OR (95% CI)**	**aOR (95% CI)**
	Yes = 459	No = 670		
Variables	n (%)	n (%)		
Spoken to HCP about vaccine during perinatal period	331 (66.6)	215 (32.1)	4.22 (3.30–5.40)	3.48 (2.61–4.63)
Beliefs: Understand how vaccine works	405 (81.5)	486 (72.5)	1.67 (1.26–2.21)	1.17 (0.81–1.70)
Beliefs: Believe vaccines are effective	459 (92.4)	546 (81.5)	2.74 (1.87–4.03)	2.65 (1.61–4.34)
Worries: Infertility	46 (9.26)	110 (16.4)	0.52 (0.36–0.75)	0.59 (0.34–1.01)
Worries: Pregnancy loss	45 (9.1)	86 (12.8)	0.68 (0.46–0.99)	1.92 (1.06–3.49)
Worries: Fetal growth and development problems	67 (13.5)	153 (22.8)	0.53 (0.39–0.72)	0.53 (0.30–0.93)
Worries: Child behavioural neurodevelopmental problems	58 (11.7)	131 (19.6)	0.54 (0.39–0.76)	0.65 (0.37–1.16)
Date of survey completion			1.05 (1.04–1.06)	1.05 (1.05–1.06)

^a^Date of survey completion is entered as a continuous variable to account for variability in the availability of the vaccine or changes to COVID-19 related policies

Table 3 presents factors associated with willingness to receive the COVID-19 vaccine among unvaccinated individuals, stratified by reproductive status. Among all respondents, the factors positively associated with willingness were speaking to a HCP about the vaccine during the perinatal period (aOR: 1.67, 95%CI: 1.32–2.12) and believing the COVID-19 vaccine is effective (aOR: 3.56, 95%CI: 2.70–4.69). Factors negatively associated with willingness were infertility concerns (aOR: 0.66, 95%CI: 0.49–0.88), fetal growth and development (aOR: 0.33, 95%CI: 0.24–0.46), and future child behavioral neurodevelopmental problems (aOR: 0.64, 95%CI: 0.48–0.84). Similar patterns were found across each sub-group.

**Table 3:** Factors associations with willingness to get the COVID-19 vaccine among unvaccinated people (*n* = 1982) and sub-group analyses among preconception, pregnant, and breastfeeding individuals

**A. Total (*n* = 1982)**	**Willing to be vaccinated**			**OR (95% CI)**		**aOR (95% CI)**
	Yes = 1447	No = 539				
Variables	n (%)	n (%)				
Spoken to HCP about vaccine during perinatal period	835 (57.7)	268 (49.7)	1.38 (1.13–1.68)		1.67 (1.32–2.12)	
Beliefs: I have a good understanding of how the COVID-19 vaccine works	1077 (74.4)	328 (60.9)	1.87 (1.52–2.31)		0.89 (0.69–1.15)	
Beliefs: COVID-19 vaccine is effective	1275 (88.1)	325 (60.3)	4.88 (3.86–6.17)		3.56 (2.70–4.69)	
Worries: May lead to infertility	188 (13.0)	211 (39.2)	0.23 (0.18–0.29)		0.66 (0.49–0.88)	
Worries: May lead to pregnancy loss or stillbirth	398 (27.5)	302 (56.0)	0.30 (0.24–0.37)		0.85 (0.65–1.13)	
Worries: May lead to fetal growth and development problems	527 (36.4)	411 (76.3)	0.18 (0.14–0.22)		0.33 (0.24–0.46)	
Worries: Child behavioural neurodevelopmental problems	409 (28.2)	336 (62.3)	0.24 (0.19–0.29)		0.64 (0.48–0.84)	
Date of survey completion^a^			0.98 (0.98–0.98)		0.99 (0.98–0.99)	
**B. Pre-conception (*n* = 254)**	**Willing to be vaccinated**			**OR (95% CI)**		**aOR (95% CI)**
	Yes = 171	No = 83				
Variables	n (%)	n (%)				
Spoken to HCP about vaccine during perinatal period	59 (34.5)	27 (32.5)	1.10 (0.63–1.91)		1.44 (0.75–2.74)	
Beliefs: I have a good understanding of how the COVID-19 vaccine works	119 (69.6)	47 (56.6)	1.75 (1.02–3.02)		0.83 (0.43–1.58)	
Beliefs: COVID-19 vaccine is effective	146 (85.4)	46 (55.4)	4.70 (2.56–8.61)		3.48 (1.76–6.88)	
Worries: May lead to infertility	65 (38.0)	62 (74.7)	0.21 (0.12–0.37)		0.45 (0.22–0.93)	
Worries: May lead to pregnancy loss or stillbirth	94 (55.0)	69 (83.1)	0.25 (0.13–0.47)		0.50 (0.21–1.20)	
Worries: May lead to fetal growth and development problems	108 (63.2)	73 (88.0)	0.24 (0.11–0.49)		0.50 (0.18–1.38)	
Worries: Child behavioural neurodevelopmental problems	85 (49.7)	58 (69.9)	0.43 (0.24–0.74)		1.07 (0.49–2.35)	
Date of survey completion			0.98 (0.97–0.99)		0.99 (0.98–1.01)	
**C. Pregnancy (*n* = 1062)**	**Willing to be vaccinated**			**OR (95% CI)**		**aOR (95% CI)**
	Yes = 716	No = 346				
Variables	n (%)	n (%)				
Spoken to HCP about vaccine during perinatal period	592 (82.7)	210 (60.7)	3.09 (2.31–4.13)		3.34 (2.39–4.67)	
Beliefs: I have a good understanding of how the COVID-19 vaccine works	533 (74.4)	220 (63.6)	1.67 (1.27–2.20)		0.84 (0.60–1.13)	
Beliefs: COVID-19 vaccine is effective	636 (88.8)	226 (65.3)	4.22 (3.06–5.82)		2.79 (1.19–4.06)	
Worries: May lead to infertility	62 (8.7)	100 (28.9)	0.233 (0.16–0.33)		0.55 (0.36–0.83)	
Worries: May lead to pregnancy loss or stillbirth	254 (35.5)	197 (56.9)	0.42 (0.32–0.54)		1.07 (0.76–1.51)	
Worries: May lead to fetal growth and development problems	326 (45.5)	278 (80.4)	0.20 (0.15–0.28)		0.33 (0.22–0.50)	
Worries: Child behavioural neurodevelopmental problems	244 (34.1)	227 (65.6)	0.27 (0.21–0.36)		0.61 (0.43–0.86)	
Date of survey completion			0.99 (0.98–0.99)		0.99 (0.98–1.00)	
**D. Breastfeeding (*n* = 670)**	**Willing to be vaccinated**			**OR (95% CI)**		**aOR (95% CI)**
	Yes = 560	No = 110				
Variables	n (%)	n (%)				
Spoken to HCP about vaccine during perinatal period	184 (32.9)	31 (28.2)	1.25 (0.79–1.96)		1.40 (0.82–2.40)	
Beliefs: I have a good understanding of how the COVID-19 vaccine works	425 (75.9)	61 (55.5)	2.53 (1.66–3.86)		1.01 (0.58–1.75)	
Beliefs: COVID-19 vaccine is effective	493 (88.0)	53 (48.2)	7.91 (5.03–12.45)		5.91 (3.43–10.19)	
Worries: May lead to infertility	61 (10.9)	49 (44.6)	0.15 (0.10–0.24)		0.44 (0.23–0.83)	
Worries: May lead to pregnancy loss or stillbirth	50 (8.9)	36 (32.7)	0.20 (0.12–0.33)		0.71 (0.34–1.47)	
Worries: May lead to fetal growth and development problems	93 (16.6)	60 (54.6)	0.17 (0.11–0.26)		0.51 (0.25–1.04)	
Worries: Child behavioural neurodevelopmental problems	80 (14.3)	51 (46.4)	0.19 (0.13–0.30)		0.60 (0.31–1.15)	
Date of survey completion			0.98 (0.97–0.99)		0.98 (0.97–0.99)	

^a^Date of survey completion is entered as a continuous variable to account for variability in the availability of the vaccine or changes to COVID-19 related policies

## Discussion

### Principal findings

In this Canadian perinatal survey, conducted in the early phases of the mRNA COVID-19 vaccine rollout during pregnancy, 42.4% of respondents reported having received at least one COVID-19 vaccine dose. Among unvaccinated individuals, 72.8% indicated a willingness to get vaccinated. The majority of respondents believed that COVID-19 vaccines were effective, understood how they work, and understood that antibodies could be passed between pregnant/lactating individuals and their infants. The most common worries were concerns about fetal growth and development, future behavioral and neurodevelopmental problems in the child, and pregnancy loss. Access to HCPs to discuss COVID-19 vaccination during pregnancy was the strongest factor associated with being vaccinated and believing the COVID-19 vaccine is effective was the strongest factor associated with willingness to be vaccinated among unvaccinated people.

### Results in the context of what is known

This survey study was conducted during the earlier stages of the vaccine rollout in Canada when the public had less information regarding safety, effectiveness and uptake of the COVID-19 vaccine in the perinatal population. Despite this, many had already been vaccinated and most of the unvaccinated people were willing to get vaccinated, highlighting a high vaccine acceptance. Provider recommendations, in particular, play an important role in shaping an individual’s perceptions regarding vaccination in pregnancy and a lack of recommendation and inconsistent messaging are linked to low vaccine uptake [19–21]. Other factors include an individual’s perceived magnitude of risk to themselves or the developing infant if they remain unvaccinated, and their perception regarding vaccine effectiveness and safety [21–23]. The findings of this survey are consistent with existing studies about perinatal vaccine acceptance and hesitancy in general, and for COVID-19 specifically, supporting the notion that beliefs and worries are strong motivators of individual decision-making and COVID-19 vaccination behaviour [10]..

In our study, the most common motivating factors associated with willingness to get vaccinated were supportive scientific evidence, being advised by a maternity HCP to get vaccinated, and if it was recommended by public health officials. These findings are higher than a similar large multi-country survey which found that 52% of unvaccinated pregnant individuals (n = 2,747/5,282) were willing to receive the COVID-19 vaccine [24]. The strongest predictors of vaccine acceptance in this population included confidence in vaccine safety or effectiveness, belief in the importance of vaccines, compliance with mask guidelines, trust of public health agencies and positive attitudes toward routine vaccination. Individuals who were reluctant to get vaccinated cited concerns regarding the risks of side effects of exposing their baby to the COVID-19 vaccine [24]. Our findings also support the importance of understanding and believing the vaccine is effective.

Several studies have identified the differences in vaccine acceptance comparing pregnant to non-pregnant individuals. A cross-sectional study conducted in Romania found that pregnant individuals were significantly more hesitant to receive the COVID-19 vaccine than non-pregnant individuals [13]. A study from the United Kingdom investigated pregnant individuals’ views on COVID-19 vaccine acceptability and found that COVID-19 vaccine acceptance was significantly lower during pregnancy [17]. Additionally, individuals who did not receive other perinatal vaccines (e.g., Tdap) were over four times more likely to reject COVID-19 vaccines during pregnancy, safety concerns about COVID-19 vaccines were common, and mistrust in vaccines was also expressed [17]. Trust in vaccines and the health system were also reasons for accepting COVID-19 vaccines [17]. Our study complements this literature with additional information on three perinatal groups (preconception, pregnant, lactating) focusing both on the prevalence of vaccination and willingness to be vaccinated among the unvaccinated.

### Clinical implications

Perception of susceptibility and severity of health threats have a major influence on maternal decision-making regarding vaccinations in pregnancy. The unknown risks of new vaccines, perceived ‘untested’ vaccines, and worries about vaccine safety drive decision-making related to COVID-19 vaccination [25]. Since neonates are currently ineligible to receive COVID-19 vaccines, as with influenza vaccines [26], maternal vaccination may be the only mechanisms available to provide antibody protection to the newborn.

Education and effective communication strategies can be used to address the motivational factors identified in this study [27]. Tailored messages can be developed for specific populations that focus on vaccine safety concerns and address the specific high priority worries identified in this study (e.g. fetal growth and development, future behavioural and neurodevelopmental problems in the child, and pregnancy loss). This evidence-based approach can be used to support decision-making, reduce hesitancy in pregnant and lactating individuals who are reluctant or unsure about receiving the COVID-19 vaccine during pregnancy and improve uptake. In addition to equipping HCPs with up-to-date information on perinatal vaccination, other options include vaccine consult lines to speak to a trained HCP who is up-to-date on the specific evidence in the perinatal population.

Mitigating concerns over negative side-effects of the vaccine is critical. Infertility was a major concern, and it is important for governments and professional associations to quickly address misinformation circulating online and on social media with scientific evidence. Many respondents were concerned with fetal growth and development, and there are now many studies available for other vaccines showing that vaccination during pregnancy is not associated with long-term adverse health consequences in children [28–31]. These should be shared widely and used in knowledge dissemination campaigns with HCPs and our communities. Providing data on long term safety studies on other vaccines routinely given in pregnancy could be an effective strategy when discussing concerns around potential neurodevelopmental problems for the infant in the future [8].

### Research implications

The most pressing area for continued evidence is on the long-term effects of the COVID-19 vaccine during pregnancy on the future development of the child exposed to the vaccine in utero or during lactation. This was one of the most commonly cited concerns about vaccination during pregnancy. More research is needed to explore the barriers to vaccine uptake across different populations, including vulnerable groups, and to determine the most effective approach to address different individual and contextual factors that influence vaccine acceptance and hesitancy within each group. Future research can investigate the most effective strategies to address the barriers to vaccine uptake during pregnancy and whether patient decision-aids can be used to support shared decision-making in COVID-19 vaccine counselling.

### Strengths and limitations

This study includes information from a sample of respondents in different reproductive stages (preconception, pregnant, lactating), which brings depth and nuance into considerations for each group. Additionally, the large sample contributes to lower variability in the estimates. Participants who used a new device or cleared their web browser’s cache and cookies may have submitted multiple entries. Individuals self-selected into the study, resulting in sociodemographic homogeneity (e.g., 83.0% White, 65.8% higher income, 86.1% higher education); this limits the generalizability of the findings to the broader Canadian population of preconception, pregnant and lactating individuals. Measurement bias may be present as survey items were exclusively self-reported and the questions were not derived from validated instruments. As this was a cross-sectional survey, the regression analyses produce odds and adjusted odds ratios that can only estimate associations and not causation. The survey was administered from March to August 2021, during which time recommendations, prioritization, and access to vaccines changed. This may have influenced participant-reported beliefs and uptake.

## Conclusions

This study identified that in the early phase of vaccine rollout in Canada when less than half of the perinatal population had been vaccinated, the majority of unvaccinated individuals were willing to be vaccinated. Access to maternity care providers during the perinatal period to have questions about the vaccine during pregnancy addressed and believing the COVID-19 vaccine is effective were the most important factors influencing vaccine uptake and willingness. Top concerns were regarding fetal growth and development, and neurodevelopmental problems for the infant in the future. Equipping HCPs with important vaccine safety and effectiveness information are critical to support shared decision-making during the perinatal period. Ongoing research on long-term infant and child outcomes will be important for informed decision-making, particularly as new vaccines are developed.

## Supplementary Information

**Additional file 1.** PLAN-V Questionnaire.

## Data Availability

The datasets used and/or analysed during the current study are available from the corresponding author on reasonable request.

## Abbreviations

Tdap: Tetanus-diphtheria-acellular pertussis
HCP: Healthcare providers
NACI: National Advisory Committee on Immunization
OR: Odds ratios
CI: Confidence intervals
aOR: Adjusted odds ratios
