# Supplementary material for: Worries, beliefs and factors influencing perinatal COVID-19 vaccination: a cross-sectional survey of preconception, pregnant and lactating individuals
Source: BMC Public Health. 2022 Dec 23;22:2418. doi: 10.1186/s12889-022-14617-4 (PMC9780097; doi:10.1186/s12889-022-14617-4)
Supplement: Supplementary file 1 — Additional file 1. PLAN-V Questionnaire. [file 12889_2022_14617_MOESM1_ESM.pdf]

PLAN-V: Pregnant, Lactation Women & Newborn COVID-19 Vaccination Survey

Part A: COVID-19 Vaccine status & exposure history

1. Which of the following options best describes your current pregnancy status? Select one option with which you most identify.

- Pregnant
- Not pregnant, but planning to become pregnant within the next year
- Breastfeeding
- None of the above ***these participants will be excluded from participation in the survey***

a) *Pregnant:* Which trimester?

- 1st (1-13 weeks)
- 2nd (14-27 weeks)
- 3rd (28-40 weeks)

b) *Breastfeeding:* How long ago did you deliver?

- <4 weeks
- 4-8 weeks
- 9-12 weeks
- 13-24 weeks
- >24 weeks

2. Based on your lifestyle (i.e., day-to-day life, work/occupation, members of household etc.), how likely do you think you will be infected by COVID-19?
   - Very likely
   - Somewhat/Fairly likely
   - Not very likely
   - Not at all likely

3. How worried are you about the possibility of becoming infected with COVID-19?

   - Very worried
   - Fairly worried
   - Not very worried
   - Not at all worried

4. How worried are you about the possibility of someone else in your household or other close contact becoming infected with COVID-19?

- Very worried
- Fairly worried
- Not very worried
- Not at all worried

5. If you become infected with COVID-19, how likely do you think you are to become seriously ill?

   - Very likely
   - Somewhat/fairly likely
   - Not very likely
   - Not at all likely

6. *Pregnant and pre-conception:* If you become infected with COVID-19, how likely do you think that infection with COVID-19 will impact your pregnancy (e.g., early delivery and other pregnancy complications)?

   - Very likely
   - Somewhat/fairly likely
   - Not very likely
   - Not at all likely

7. *Pregnant and pre-conception:* How likely do you think it will impact the health of your fetus/baby while pregnant?

   - Very likely
   - Somewhat/fairly likely
   - Not very likely
   - Not at all likely

8. To your knowledge, are you, or have you been infected with COVID-19?

   - Yes
     - Was it mild or sever?
       - Mild
       - Moderate
       - Severe
     - Was it confirmed by a test?
       - Confirmed by a test

        - Not confirmed by a test
- No

9. Have you been vaccinated against COVID-19? Answer 'Yes' if you received at least one dose of the COVID-19 vaccine.

   - Yes
     - How many doses of the COVID-19 vaccine have you received so far?
       - One dose
         - When did you receive your first dose of the COVID-19 vaccine?
       - Two doses
         - When did you receive your second dose of the COVID-19 vaccine?
   - No

10. *If ' Yes' to vaccinated against COVID-19:* Which vaccine(s) did you receive? Select all that apply.

    - Pfizer and BioNTech mRNA vaccine
    - Modern mRNA vaccine
    - AstraZeneca Oxford vaccine
    - Janssen (Johnson and Johnson) vaccine
    - Other – please specify
    - I don't know

11. *If ' Yes' to vaccinated against COVID-19:* At the time of your 1st and/or 2nd COVID-19 vaccine, were you:

    - Thinking of trying to get pregnant in the next 6-12 months
    - Trying to get pregnant
    - Pregnant
      - In which trimester did you receive your 1st dose?

        - 1st (1-13 weeks)
        - 2nd (14-27 weeks)
        - 3rd (28-40 weeks)

    - Breastfeeding
    - None of the above

12. *If ' Yes' to vaccinated against COVID-19:* Why did you decide to get vaccinated against COVID-19? Select all that apply.

- My family doctor advised that I should receive it
- My maternity care provider (e.g., obstetrician, midwife, family medicine obstetrics provider) advised that I should receive it
- my friends and/or family recommended it to me
- another pregnant person recommended it to me
- public health officials recommended the vaccine for pregnant and breastfeeding individuals
- the scientific evidence proved it was safe during pregnancy and breastfeeding
- I am at high risk of contracting COVID-19
- I am at risk of more severe symptoms and/or outcomes after contracting COVID-19
- To prevent myself from getting COVID-19 or from becoming seriously ill
- To prevent my baby from getting COVID-19 or from becoming seriously ill
- I believe I would be doing my part in fighting against COVID-19 by contributing to herd immunity/herd protection and by making COVID-19 harder to spread and replicate
- Other – please specify:

Part B: Medical History

1. Do you have any of the following health conditions? Select all that apply.

- Diabetes (Type 1, Type 2, gestational diabetes)
- Hypertension (high blood pressure)
- Higher body weight
- Thyroid disease
- Autoimmune disease (conditions that effect your immune system, e.g., lupus, arthritis, Crohn’s disease, etc.)
- Immune suppressed
- Respiratory conditions (conditions that effect your breathing and lungs, e.g., asthma, etc.)
- Current or previous diagnoses of cancer
- Severe allergies (anaphylactic, hives/welts, red rash, swollen throat or swollen areas of the body, pale or red colour to the face and body)
- Not applicable
- Other, please specify:

2. In the past year, how many times were you treated by a healthcare provider (<u>not</u> including prenatal/obstetrical care or well-baby visits)?

- 0 times
- 1-4 times
- 5-10 times

- > 10 times
- Unsure

3. *Pregnant*: How many prenatal care visits have you attended in your current pregnancy (including in-person and/or virtual visits)?

- I attended all of my scheduled prenatal appointments
- I attended most of my scheduled prenatal appointments
- I attended some of my scheduled prenatal appointments
- I did not attend any prenatal care visits
- Unsure

4. *Breastfeeding:* How many prenatal care visits did you attend in your most recent pregnancy (including in-person and/or virtual visits)?

- I attended all of my scheduled prenatal appointments
- I attended most of my scheduled prenatal appointments
- I attended some of my scheduled prenatal appointments
- I did not attend any prenatal care visits
- Unsure

5. *Pregnant:* From which type of healthcare provider are you currently receiving the majority of your prenatal care?

- Obstetrician
- Family physician
- Midwife
- Other, please specify:

6. *Breastfeeding:* From which type of healthcare provider did you receive the majority of your prenatal care for your most recent pregnancy?

- Obstetrician
- Family physician
- Midwife
- Other, please specify:

7. Have you spoken with a healthcare provider about receiving the COVID-19 vaccine while trying to get pregnant, or during pregnancy, or breastfeeding?

   - Yes
     - Who did you speak with? Select all that apply.
       - Obstetrician
       - Family physician
       - Midwife

        - Nurse
        - Family/Friend who is a healthcare provider
        - Other – please specify
    - Did they recommend getting the COVID-19 vaccine while trying to get pregnant, or during pregnancy, or breastfeeding?
        - Yes
        - No
- No

8. How many times have you been pregnant (including current pregnancy)? This includes any type of loss (e.g., miscarriage or abortion) before 20 weeks of pregnancy.

- 0 (*if women select this option, they will be prompted to question 8*)
- 1
- 2
- 3+

9. *Participants with previous pregnancy:* How many pregnancies have you had that you delivered after 20 weeks of pregnancy (excluding most recent or current pregnancy)?

- 0
- 1
- 2
- 3+

10. *Participants with previous pregnancy:* How many times have you delivered a live baby (excluding most recent or current pregnancy)?

- 0
- 1
- 2
- 3+

11. *Breastfeeding:* What feeding methods are you currently using, or did you use to feed your infant during the first six months after your most recent pregnancy? Select all that apply.

- Breast milk
- Formula or milk supplements
- Solid food
- Other – please specify:

12. *Pregnant and Breastfeeding:* Did you receive any vaccines during your current or most recent pregnancy?

- Yes
    - If yes, please select all that apply.
        - Flu shot/Influenza vaccine
            - Please select the trimester of pregnancy that you received the vaccine:
                - 1st (1-13 weeks)
                - 2nd (14-27 weeks)
                - 3rd (28-40 weeks)
        - Tdap/Pertussis/Adacel vaccine
            - Please select the trimester of pregnancy that you received the vaccine:
                - 1st (1-13 weeks)
                - 2nd (14-27 weeks)
                - 3rd (28-40 weeks)
        - Other – please specify the vaccine and the trimester:
            - Were these vaccines recommended by your healthcare provider?
                - Yes
                - No
- No
- Unsure

13. *Participants with previous live birth*: Have you ever decided **<u>not</u>** to vaccinate your child(ren) for reasons other than illness or allergy?

- No
- Yes
    - Are there any vaccine types/brands in particular?
        - No, all vaccines
        - Yes, please specify:
    - Please indicate reason why:

14. If you gave birth to a child today, and they had no illness or allergies, would you choose to keep your child up to date with all recommended vaccines?

- Yes
- No
    - Are there any vaccine types/brands in particular?
        - No, all vaccines
        - Yes, please specify:
    - Please indicate reason why:

Part C: Intentions to Receive COVID-19 Vaccination

1. *Pre-conception:* When would you be willing to receive the COVID-19 vaccine? If you have already received the COVID-19 vaccine, please answer hypothetically. Select all that apply.

– Pre-conception (pre-pregnancy)
– During pregnancy
– While breastfeeding
– After delivery, while not breastfeeding
– After having all planned children
– I would never be willing to receive the COVID-19 vaccine (*will be prompted to answer question 4*)

2. *Pregnant:* When would you be willing to receive the COVID-19 vaccine? If you have already received the COVID-19 vaccine, please answer hypothetically. Select all that apply.

– During pregnancy
– While breastfeeding
– After delivery, while not breastfeeding
– After having all planned children
– I would never be willing to receive the COVID-19 vaccine (*will be prompted to answer question 4*)

3. *Breastfeeding:* When would you be willing to receive the COVID-19 vaccine? If you have already received the COVID-19 vaccine, please answer hypothetically. Select all that apply.

– While breastfeeding
– After delivery, while not breastfeeding
– After having all planned children
– I would never be willing to receive the COVID-19 vaccine (*will be prompted to answer question 4*)

4. *If participant answered 'I would not be willing to receive the COVID-19 vaccine'*: Please complete the following sentence by selecting all that apply. "I would not receive the COVID-19 vaccine because…"

– I'm not eligible due to illness or allergy
– I have a fear of needles
– there is a lack of information on safety in pregnant and breastfeeding persons
– there is a lack of information about possible long-term health effects
– I have concerns about the risks and side effects to myself
– I have concerns about the risks during pregnancy
– I have concerns about the risks to my fertility or my ability to get pregnant

- I have concerns about the effect on my fetus/baby
- I do not believe it is necessary for me to get the vaccine
- I do not believe it will protect me against COVID-19
- of my religious beliefs
- my husband/spouse/significant other prefers that I do not receive the vaccine
- I am still undecided about whether I should receive the vaccine
- I do not feel well informed enough to make a decision at this time
- Other - please specify:

Part D: Perceptions and Factors Influencing COVID-19 Vaccination

1. Please complete the following sentence by selecting all that apply. "I believe…"

- I have a good understanding of how the COVID-19 vaccine works
- the COVID-19 vaccine is an effective way to prevent COVID-19
- pregnant and breastfeeding persons would benefit from receiving the COVID-19 vaccine only if they are at high risk of becoming infected with COVID-19 (e.g., frontline worker, healthcare staff, etc.)
- pregnant and breastfeeding persons would benefit from receiving the COVID-19 vaccine only if they are at high risk of becoming severely ill with COVID-19, due to other health conditions (e.g., high blood pressure or lung conditions)
- all pregnant and breastfeeding persons would benefit from receiving the COVID-19 vaccine regardless of state of health or other risk factors
- pregnant and breastfeeding persons who receive the COVID-19 vaccine can pass protective antibodies to their babies
- None of the above
- Other - please specify:

2. Please complete the following hypothetical statement around short and long-term effects of the COVID-19 vaccine by selecting all that apply. "I am worried that if I get the COVID-19 vaccine it may lead to…"

- infertility (i.e., your ability to get pregnant in the future)
- pregnancy loss or stillbirth
- growth and development issues in my current or future unborn baby (e.g., birth defects, preterm birth)
- behavioral or neurodevelopmental changes in my child in the future (e.g., autism, attention deficit hyperactivity disorder [ADHD], learning disability)
- an increased chance of cancer or other life-threatening conditions for myself
- an increased chance of cancer or other life-threatening conditions for my baby
- I am not worried about any possible short-term or long-term side effects from the COVID-19 vaccine

- Other - please specify:

3. *Excluded those that have received vaccine:* Please complete the following statement related to individuals influencing your decision to receive the COVID-19 vaccine by selecting all that apply. Please complete the following sentence: “I would be motivated to receive the COVID-19 vaccine if…”

- my family doctor advised that I should receive it
- My primary maternity care provider (e.g., obstetrician, midwife, family medicine obstetrics provider) advised that I should receive it
- my friends and/or family recommended it to me
- another pregnant person recommended it to me
- public health officials recommended the vaccine for pregnant and breastfeeding individuals
- the scientific evidence proved it was safe during pregnancy and breastfeeding
- it was recommended by an international organization (e.g., World Health Organization)
- it would facilitate travel (i.e., to enter another country) and/or ability to attend social gatherings (i.e., concerts, sporting events, weddings, visiting older relatives etc.)
- I would never be motivated to receive the COVID-19 vaccine.
- Other - please specify:

4. From where are you receiving your COVID-19 information? Select all that apply.

- News broadcasting (e.g., television, radio, newspapers, magazines, etc.)
- Social media (e.g., Facebook, Twitter, Instagram, Snapchat)
- Health care provider(s)
- Government issued websites (e.g., Government of Canada, Public Health Ontario, Ottawa Public Health, World Health Organization, Centers for Disease Control and Prevention)
- Pregnancy and/or breastfeeding professional societies (e.g., Society of Obstetricians and Gynaecologists Canada, American College of Obstetricians and Gynecologists)
- General pregnancy and childbirth/parenting websites
- Online forums (e.g., Reddit, Quora)
- Other - please specify:

<u>PART XX: Demographic Data</u>

1. How old are you?

- <18
- 18-29
- 30-39
- 40-49
- 50-64

- 65+
- Prefer not to answer

2. What is your gender identity? Select one.

- Women
- Intersex
- Non-binary (genderqueer)
- Transgender
- Gender Fluid
- Questioning
- Two-spirit
- Man
- Do not know
- Prefer not to answer
- Other – please specify:

3. What is your sexual orientation? Select all that apply.

- Heterosexual/straight
- Homosexual (e.g., gay, lesbian, queer)
- Bisexual
- Pansexual
- Asexual
- Prefer not to answer
- Other – please specify:

4. Which race/ethnicity do you <u>most</u> identify with? Select one.

- Asian – East (e.g. Chinese, Japanese, Korean)
- Asian – South (e.g. Malaysian, Filipino, Vietnamese)
- Black – African (e.g. Ghanaian, Kenyan, Somali)
- Black – Caribbean (e.g. Barbadian, Jamaican)
- Black – North American (e.g. Canadian, American)
- First Nations Indian – Caribbean (e.g. Guyanese with origins in India)
- First Nations – origination from North American
- Indigenous/Aboriginal not included elsewhere
- Inuit

- Metis
- Latin American (e.g. Argentinean, Chilean, Salvadorian)
- Middle Eastern (e.g. Egyptian, Iranian, Lebanese)
- White – European (e.g. English Italian, Portuguese, Russian)
- White – North American (e.g. Canadian, American)
- Mixed Heritage
- Prefer not to answer
- Other, please specify:

5. What religious group do you most identify with?

- Buddhist
- Christian
  - Anglican
  - Baptist
  - Catholic
  - Christian Orthodox
  - Lutheran
  - Pentecostal
  - Presbyterian
  - United Church
  - Other Christian
- Hindu
- Jewish
- Muslim
- Sikh
- Traditional (Aboriginal) Spirituality
- No religious affiliation
- Prefer not to answer
- Other – Please specify:

6. What is your marital status?

- Single
- Married/Common law
- Separated/Divorced
- Prefer not to answer
- Other, please specify:

7. What is your living situation? Select all that apply.

- Alone
- With children
- With parents
- With other family members

- With non-family roommates
- Prefer not to answer
- Other – please specify

8. Do you currently reside in Canada?

- Yes
    - If yes, please indicate:
        - Province:
        - What is the first three digits of your postal code? (e.g., L1B):
        - Number of years you have resided in Canada:
- No
    - If no, please indicate the country:

9. In which language are you <u>most</u> fluent? (i.e., your mother tongue) Select one.

- English
- French
- Arabic
- Italian
- Spanish
- Chinese (Mandarin, Cantonese, or other Chinese dialect)
- Other:

10. What is your highest level of completed education? Select one.

- Less than high school diploma/high school equivalency
- Completed high school diploma or a high school equivalency certificate
- Trades certificate or diploma
- College, CEGEP or other non-university certificate or diploma (Exclude trades certificates or diplomas)
- University certificate or diploma below the bachelor's level
- Bachelor's degree (e.g., B.A, B.A. (hons), B. Sc., B.Ed., LL.B.)
- University certificate, diploma, or degree above the bachelor's level
- Prefer not to answer

11. Which best applies to your current employment status?

- Full-time
- Part-time
- Unemployed
- Paid or unpaid leave (includes parental leave)
- Prefer not to answer

12. What is your occupation?

- Front-facing health worker (i.e., face-to-face interaction with patients)
- Non-front-facing health worker (i.e., administrators, managers, clerical, research, etc.)
- Essential services worker (i.e., non-health frontline worker, police, transport service worker, grocery store staff, etc.)
- Educator
- Other work
- Retired
- None of the above

13. Are you a health care professional?

- Yes
    - What is your current role?
        - Doctor
        - Nurse
        - Paramedic/First responder
        - Allied health
        - Community health worker
        - Traditional healer
        - Other health worker
- No

14. What is your total household income before taxes?

- Less than $30,000
- $30,000 to $59,999
- $60,000 to $89,999
- $90,000 to $119,999
- $120,000 to $149,999
- More than $150,000
- Prefer not to answer

15. How did you hear about this survey?

- Social media (e.g., Facebook, Twitter, Instagram)
- Healthcare provider referral
- Flyer/poster
- Other - please specify:

16. Please select the date you are completing this survey:

17. Thank you for completing the survey. If you have any other feedback about COVID-19 vaccination during pregnancy, breastfeeding or pre-conception period, please enter them below:
